# Does High-Intensity Exercise Combined with Exposure Sessions Improve Treatment Outcomes in Obsessive-Compulsive Disorder? A Randomized Controlled Trial in Inpatients

**DOI:** 10.1159/pps/aemag016

**Published:** 2026-09-30

**Authors:** Mark Mühlbacher, Rebecca Schennach, Thomas Bösl, Christoph Bamberg, Stefan Koch, Ulrich Voderholzer

**Affiliations:** a Department of Psychiatry and Psychotherapy, LMU University Hospital, LMU Munich, Munich, Germany; b Department of Psychiatry and Psychotherapy, University Hospital of Freiburg, Freiburg, Germany; c Schoen Clinic Roseneck, Prien am Chiemsee, Germany; d Division of Clinical Psychology and Health Psychology, Department of Psychology, University of Salzburg, Salzburg, Austria

**Keywords:** Obsessive-compulsive disorder, Behavioral exposure, Aerobic training, Adjunctive treatment, Clinical trial

## Abstract

**Introduction:**

Physical exercise administered before or after exposure has been shown to have beneficial effects on treatment outcomes in anxiety- and trauma-related disorders. However, this approach has not yet been examined for obsessive-compulsive disorder (OCD). This randomized controlled trial investigated whether high-intensity exercise administered immediately after exposure therapy improves treatment outcomes in patients with OCD.

**Methods:**

Ninety-seven inpatients with OCD participated in an 8-week exposure-based treatment phase during inpatient care, comprising 2 sessions per week, and were randomly assigned to either high-intensity exercise (ExpoHI) or low-intensity control (ExpoLI) administered immediately after a standardized exposure and response prevention session. OCD severity was assessed using the Yale-Brown Obsessive Compulsive Scale (Y-BOCS) at admission, discharge, and 6-month follow-up. The secondary outcomes included general psychopathological symptoms.

**Results:**

ExpoHI showed significantly greater reductions in Y-BOCS scores at discharge (between-group difference: −3.14 points, *p* = 0.009), corresponding to a moderate-to-large between-group effect size (Cohen’s *d* = 0.76), which was maintained at the 6-month follow-up (*p* = 0.030; Cohen’s *d* = 0.68). Responder rates at discharge were significantly higher in the ExpoHI group (85.4%) than in the ExpoLI group (61.2%) (*p* = 0.011), whereas remission rates did not differ between groups. ExpoHI also showed greater reductions in depressive symptoms at follow-up.

**Conclusions:**

High-intensity exercise immediately following exposure therapy produced greater reductions in obsessive-compulsive symptoms than the active control condition. These findings suggest that exercise may be a feasible adjunct for exposure-based treatment.

## Introduction

Aerobic exercise has been identified as an effective intervention alongside exposure-based treatment for anxiety disorders, with high-intensity exercise sessions consistently demonstrating greater efficacy than low-intensity aerobic activity [1]. Evidence from studies in post-traumatic stress disorder and panic disorder further suggests that coupling exposure therapy with immediate pre- or post-session aerobic exercise may enhance treatment outcomes [2, 3]. Against this background, obsessive-compulsive disorder (OCD) represents a particularly relevant clinical condition, in which strategies that enhance exposure-based treatment may further improve outcomes [4]. OCD is a severe mental health condition associated with pronounced distress and significant impairment of daily functioning [5, 6].

Exposure and response prevention (ERP) is recognized as the gold-standard treatment for OCD [7, 8] and is recommended as psychotherapy in international clinical guidelines, such as those issued by the German S3 guidelines [9] and earlier practice guidelines from the American Psychiatric Association (APA) [10]. Nevertheless, a substantial proportion of patients do not achieve satisfactory treatment response to CBT including ERP, and some experience recurrent or relapsing symptom courses [11–13]. The nonresponder rates remain relatively stable over time, and several factors have been identified as potential contributors, including insufficient treatment response [14].

Therefore, several approaches have been developed in recent years to further enhance the effectiveness of ERP, including novel treatment formats such as the Bergen 4-Day treatment [15, 16] or alternative cognitive models such as inference-based cognitive behavioral therapy [17]. In addition, inpatient treatment settings allow the delivery of high-intensity and highly structured exposure interventions that have been associated with substantial reductions in OCD symptom severity, particularly in severe treatment-resistant cases [18, 19]. However, randomized controlled trials found that augmenting ERP with acceptance and commitment therapy or mindfulness-based therapy did not provide additional benefits over ERP alone [20, 21].

Another approach to improving treatment outcomes is the addition of physical exercise as a supplementary intervention in the treatment regimen. A recent systematic review and meta-analysis of six studies (*N* = 92), including two randomized controlled trials, found that exercise interventions, including aerobic exercise, walking, resistance training, and yoga, delivered as acute or longer-term programs, were associated with large reductions in OCD symptom severity and moderate improvements in anxiety and depressive symptoms [22]. However, the authors also highlighted the considerable heterogeneity of the interventions, precluding conclusions regarding the optimal exercise modality, dose, and timing of exercise. Notably, the only study to evaluate the longer-term effects of exercise in OCD reported sustained reductions in OCD symptom severity at a 6-month follow-up following a 12-week moderate-intensity aerobic exercise intervention, although the findings were derived from an uncontrolled pilot study [23]. In a pilot randomized controlled trial, Abrantes et al. [24] evaluated a 12-week moderate-intensity aerobic exercise program consisting of weekly supervised sessions supplemented by home-based exercise as an adjunct to standard OCD treatment. Although exercise was not temporally linked to exposure sessions, the intervention resulted in a trend toward higher treatment response without significantly outperforming an active psychoeducation control. A subsequent secondary analysis showed that individual exercise sessions produced immediate, transient improvements in mood and anxiety as well as acute reductions in compulsions, although not obsessive thoughts [25]. Whether exercise administered immediately before or after exposure enhances treatment outcomes in OCD therefore remains largely unexplored.

Experimental evidence suggests that both moderate- and high-intensity exercise can improve executive functioning immediately after exercise, although improvements following high-intensity exercise may persist longer during recovery, potentially creating a transient window of enhanced cognitive processing [26]. One potential mechanism underlying these effects is that aerobic exercise may enhance extinction learning and facilitate both the retrievability and generalization of extinction memories by modulating neurobiological processes relevant to fear-related disorders and long-term cognitive functioning [27, 28]. A central factor in this context is brain-derived neurotrophic factor, which plays a key role in synaptic signaling, neuronal plasticity, neuronal survival, neuroinflammation, and neurogenesis [29, 30].

The primary aim of this study was to investigate whether the addition of high-intensity aerobic exercise following disorder-specific exposure therapy improves obsessive-compulsive symptom outcomes compared with an active post-exposure control condition. The secondary objectives were to examine group differences in depressive and anxiety symptoms and to explore response and remission rates as well as changes in physiological exercise parameters in the experimental group.

## Methods

### Design and Patients

This study was a two-arm, parallel-group superiority randomized controlled trial conducted under naturalistic inpatient conditions at a hospital for intensive treatment of OCD, Schoen Clinic Roseneck, Germany. The trial is reported in accordance with the CONSORT guidelines (see CONSORT checklist in online supplementary file; for all online suppl. material, see https://doi.org/10.1159/pps/aemag016). Participants were allocated to either a high-intensity aerobic exercise (ExpoHI) or low-intensity activity control condition (ExpoLI). Recruitment and intervention were conducted between August 2024 and September 2025. Eligible participants were adult inpatients (≥18 years) with a primary diagnosis of OCD (ICD-10 F42.2), sufficient proficiency in German, the ability to participate in the intervention sessions, and written informed consent. The exclusion criteria were as follows: a psychiatric disorder other than OCD constituting the primary focus of inpatient treatment, acute suicidality or psychosis, pregnancy, severe somatic conditions contraindicating high-intensity exercise, body mass index <17.5 kg/m^2^. Participants with hoarding disorder as the primary diagnosis were excluded. However, hoarding symptoms or comorbid hoarding disorder were not exclusion criteria if OCD was the primary diagnosis. OCD symptom subtypes were not systematically documented. The psychopharmacological treatment was managed independently by responsible ward psychiatrists according to routine clinical care, including insufficient therapeutic response, tolerability issues, or optimization based on therapeutic drug monitoring.

### Randomization

The participants were randomized in a 1:1 ratio using a computer-generated simple randomization sequence. Allocation was centrally implemented by the hospital’s Department of Therapy Coordination, and the allocation sequence remained concealed from investigators involved in participant recruitment and enrollment until treatment assignment had been completed. Owing to the nature of the intervention, participants and therapists could not be blinded to treatment allocation. Participants were informed that two adjunctive post-exposure interventions were being compared, but no specific superiority hypothesis was disclosed. Statistical analyses were performed using pseudonymized data.

### Sample Size Calculation

The sample size was calculated using G*Power 3.1 software [31]. Assuming a medium effect size (Cohen’s *d* = 0.70), α = 0.05, and power = 0.80 for a two-group repeated-measures design with three assessment points, the required total sample size was *N* = 82. The assumed effect size was based on previous randomized controlled trials investigating aerobic exercise administered in temporal conjunction with exposure-based treatment [2, 3].

### Procedure

All patients included in the study were treated on the OCD specialty ward. The participants underwent a comprehensive baseline assessment, including a structured psychiatric evaluation conducted by experienced psychiatrists according to ICD-10 criteria and a routine somatic examination, to confirm the diagnosis of OCD and eligibility for inpatient treatment and high-intensity exercise. Assessments were performed at admission (T1), discharge (T2), and 6-month follow-up (T3) using standardized online self-report measures, as specified in the publicly available preregistered study protocol. These measures were administered consistently across all assessment time points through the clinic’s routine outcome monitoring. No clinician-administered Y-BOCS or separate measure of functional outcomes was collected. Participants allocated to the experimental condition completed an Incremental Exercise Test (IET) at baseline to determine individualized training intensity. The IET was performed on a motion cycle 600 med bicycle ergometer using a standardized stepwise protocol with three-minute stages starting at 25 watts and increasing in 25-watt increments. The heart rate and performance were continuously recorded. Blood lactate concentrations were analyzed using a BIOSEN C-Line lactate analyzer. Lactate performance curves were analyzed according to the Dickhuth model [32] to determine the individual aerobic and anaerobic (IANS) thresholds. The training intensity during ExpoHI was prescribed individually based on the IANS.

### Treatment Conditions

All participants entered a structured 8-week ERP phase and received standardized twice-weekly group ERP sessions (50 min, 6–8 patients per group). Participants from both treatment conditions attended the same ERP groups. Sessions were delivered by licensed clinical psychologists or advanced psychotherapists in training with experience in OCD treatment. Overall, at least 10 therapists were involved in treatment delivery, all of whom received regular supervision by senior OCD specialists throughout ERP care. The interval between the end of the ERP session and the start of the exercise or control activity was typically approximately 5 min and only occasionally slightly longer. This short interval was facilitated by fixed therapy scheduling and pre-arranged access to the exercise equipment, allowing the intervention to commence as soon as practically feasible after ERP. ExpoHI participants then completed 25 min of high-intensity continuous aerobic cycling, including a 5-min warm-up followed by 20 min at the individually prescribed training intensity, supervised by qualified sports scientists. The workload on the cycle ergometer (watts) was individually set according to each participant’s power output at the IANS, as determined during baseline incremental exercise testing. ExpoLI participants engaged in a matched 25-min supervised group session in a separate room. Participants were instructed to engage exclusively in seated activities and could choose between board games or listening to music or podcasts while remaining focused on the selected activity throughout the session. Physical activity was explicitly prohibited. The control condition was designed to match the exercise intervention in terms of time, attention, and group setting while avoiding substantial physiological strain. To ensure a standardized implementation, the intervention was delivered at the same scheduled time each evening. No further therapeutic activities were scheduled for the remainder of the evening, allowing for the uninterrupted delivery of the assigned post-exposure intervention.

### Measures

#### Obsessive-Compulsive Symptoms

The Yale-Brown Obsessive Compulsive Scale (Y-BOCS-SR) was used as the primary outcome measure for OCD symptom severity [33, 34]. Participants completed the Y-BOCS in a self-report format after a standardized introduction. The scale comprises 10 items with total scores ranging from 0 to 40, with higher scores indicating greater symptom severity with well-established validity and reliability.

#### General Psychopathological Symptoms

Broader psychopathological symptoms were assessed using two complementary self-report instruments: the Brief Symptom Inventory (BSI) and Patient Health Questionnaire (PHQ-D) [35, 36]. The BSI comprises 53 items that evaluate subjective distress caused by various symptom dimensions. The PHQ-D assesses the severity of common mental disorders, and modules for depression, generalized anxiety, and somatic symptoms were used in this study. The BSI and PHQ-D have demonstrated good psychometric properties, including appropriate internal consistency (α > 0.80) and strong construct validity in previous validation studies [35, 36]. Both instruments were administered at the time of admission and discharge.

#### Depressive Symptoms

Depressive symptoms were assessed using the Beck Depression Inventory-II (BDI-II), a widely used 21-item self-report measure of depressive symptom severity [37, 38]. The BDI-II has demonstrated excellent internal consistency (α = 0.90–0.93) and high convergent validity in previous studies.

#### Responder and Remission Status

Treatment response and remission were derived from the Y-BOCS total scores at admission, discharge, and 6-month follow-up. Following established consensus criteria [39–41], response was defined as ≥35% reduction from baseline and remission as Y-BOCS ≤12, corresponding to minimal symptoms.

#### Exercise Parameters

Physiological exercise parameters were assessed in the experimental group at baseline and discharge using the Incremental Exercise Test. These included power output (watts), heart rate, and blood lactate concentration at the IANS, as well as maximal relative power output (W/kg).

### Statistical Analysis

Analyses were conducted using R software (version 4.5.1). The primary outcome of OCD symptom severity (Y-BOCS) was analyzed using linear mixed-effects models with time, condition, and their interaction as fixed effects and random intercepts. Model-based contrasts were used to estimate between-group differences in change from baseline, and effect sizes (Cohen’s *d*) with 95% confidence intervals were calculated. Secondary continuous outcomes were analyzed using analogous linear mixed-effects models with time, treatment condition, and their interaction as fixed effects and random intercepts for participants. Depending on the outcome, models included either two (admission, discharge) or three (admission, discharge, 6-month follow-up) assessment time points according to the predefined assessment schedule. Model assumptions were evaluated by visual inspection of residual-versus-fitted plots and normal Q-Q plots of the residuals. No relevant deviations from the model assumptions were observed. Responder and remission rates were compared using Fisher’s exact test. Analyses followed the intention-to-treat principle, meaning that all randomized participants were analyzed according to their originally assigned conditions. Missing data were handled using maximum likelihood estimation within mixed models under the missing-at-random assumption, and categorical outcomes were analyzed using the available cases. Exercise parameters were analyzed in an exploratory manner within the experimental group. Additional sensitivity analyses were conducted by adjusting the primary mixed-effects model for changes in psychopharmacological medication during treatment.

## Results

### Sample Characteristics

A total of 97 inpatients with OCD were randomized to the ExpoHI (*n* = 48) or ExpoLI (*n* = 49) groups (Fig. 1). The baseline demographic and clinical characteristics were comparable between the groups (Table 1), and no significant between-group differences were observed. The mean baseline Y-BOCS scores indicated severe obsessive-compulsive symptoms in both groups (ExpoHI: M = 30.31, SD = 4.32; ExpoLI: M = 30.39, SD = 5.02). Participants attended a mean of 13.71 (SD = 3.67) of the 16 planned sessions comprising ERP followed by the assigned post-exposure intervention, corresponding to 85.7% of all scheduled sessions. Attendance was comparable between treatment conditions (ExpoHI: M = 14.00, SD = 3.45; ExpoLI: M = 13.43, SD = 3.89; t[95] = 0.77, *p* = 0.446, Cohen’s *d* = 0.16). At discharge, outcome data were available for all 97 participants for the primary outcome and for 91–94 participants for the secondary self-report measures, depending on the instrument. At 6-month follow-up, Y-BOCS data were available for 78.4% of participants (*n* = 76; 38 per group).

**Fig. 1.**
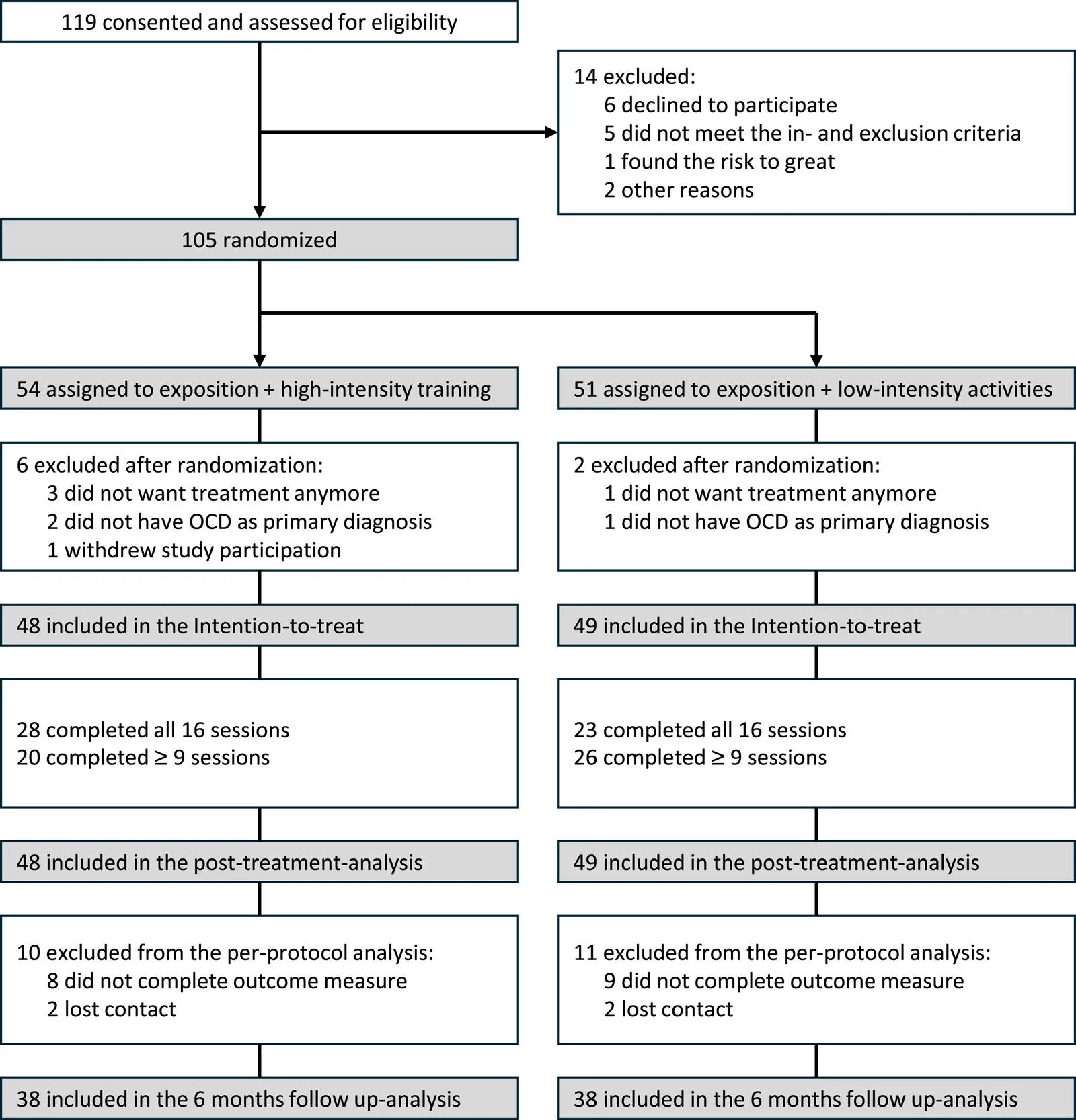
Flowchart of participants with OCD through the 8-week randomized controlled trial comparing exposure plus high-intensity exercise (ExpoHI) with exposure plus low-intensity control activities (ExpoLI).

**Table 1.** Demographics, clinical characteristics, and physiological parameters of the ITT sample

Variable	ExpoHI (*n* = 48)	ExpoLI (*n* = 49)
*Demographics*		
Age, M±SD, years	36.6±14.0	33±14.9
Sex, female	52 (25)	55 (27)
Education		
Vocational training	40 (19)	29 (14)
University degree	38 (18)	33 (16)
Partner	48 (23)	43 (21)
Children	23 (11)	20 (10)
*Clinical characteristics*		
Length of stay, M±SD, days	91.5±30.3	91.2±27.1
Prior ERP treatment before admission		
Outpatient	65 (31)	59 (29)
Inpatient	46 (22)	31 (15)
Current comorbidities		
Depressive disorder	58 (28)	69 (34)
Anxiety disorder	4 (2)	4 (2)
Somatoform disorder	4 (2)	12 (6)
Personality disorder	21 (10)	27 (13)
Eating disorder	4 (2)	12 (6)
ADHD	25 (12)	16 (8)
Number of comorbidities		
None	27 (13)	20 (10)
1	38 (18)	35 (17)
2	27 (13)	35 (17)
3+	8 (4)	10 (5)
Current medication		
None	29 (14)	33 (16)
Antidepressants	60 (29)	59 (29)
Atypical antipsychotics	23 (11)	22 (11)
Stimulant medication	13 (6)	10 (5)
Medication change^a^		
Overall	19 (9)	22 (11)
Initiation/increase	10 (5)	18 (9)
Reduction/discontinuation	8 (4)	4 (2)
*Physiological parameters*		
Body weight, M±SD, kg	73.3±15.3	71.3±18.0
BMI, M±SD, kg/m^2^	23.8±4.6	23.8±5.9
IANS^b^		
Power, M±SD, Watt	109.4±34.0	–
Lactate, M±SD, mmol/L	3.8±3.9	–
Heart rate, M±SD, bpm	138.0±15.8	–
Maximal, M±SD, W/kg	2.2±0.6	–

Data are presented as % (*n*) or means ± SD. There were no statistically significant differences between the treatment groups.

ERP, exposure and response prevention; IANS, individual anaerobic threshold (Dickhuth); BMI, body mass index; ADHD, attention-deficit/hyperactivity disorder.

^a^ Including antidepressants and atypical antipsychotics.

^b^ *n* = 2 missing.

### Primary Clinical Outcomes

Across both groups, Y-BOCS scores decreased markedly over time, reflected by a significant main effect of time (F[2, 172.54] = 305.12, *p* < 0.001), indicating substantial symptom improvement from admission to discharge that was maintained at the 6-month follow-up. Importantly, a significant time × condition interaction was observed (F[2, 172.54] = 4.11, *p* = 0.018), indicating different symptom trajectories between ExpoHI and ExpoLI (Fig. 2). Model-based difference-in-differences contrasts showed significantly greater reductions in Y-BOCS scores in ExpoHI than in ExpoLI, both at discharge and at the 6-month follow-up (Table 2). Adjusted difference-in-differences contrasts continued to show significantly greater reductions in Y-BOCS scores in ExpoHI than in ExpoLI, both at discharge (Δ = −3.14, 95% CI [−5.47, −0.81], *p* = 0.009, d = 0.76) and at the 6-month follow-up (Δ = −2.81, 95% CI [−5.34, −0.27], *p* = 0.030, d = 0.68). Sensitivity analyses adjusted for psychopharmacological medication changes demonstrated that the primary time × condition interaction remained significant (F[2,172.59] = 4.12, *p* = 0.018).

**Fig. 2.**
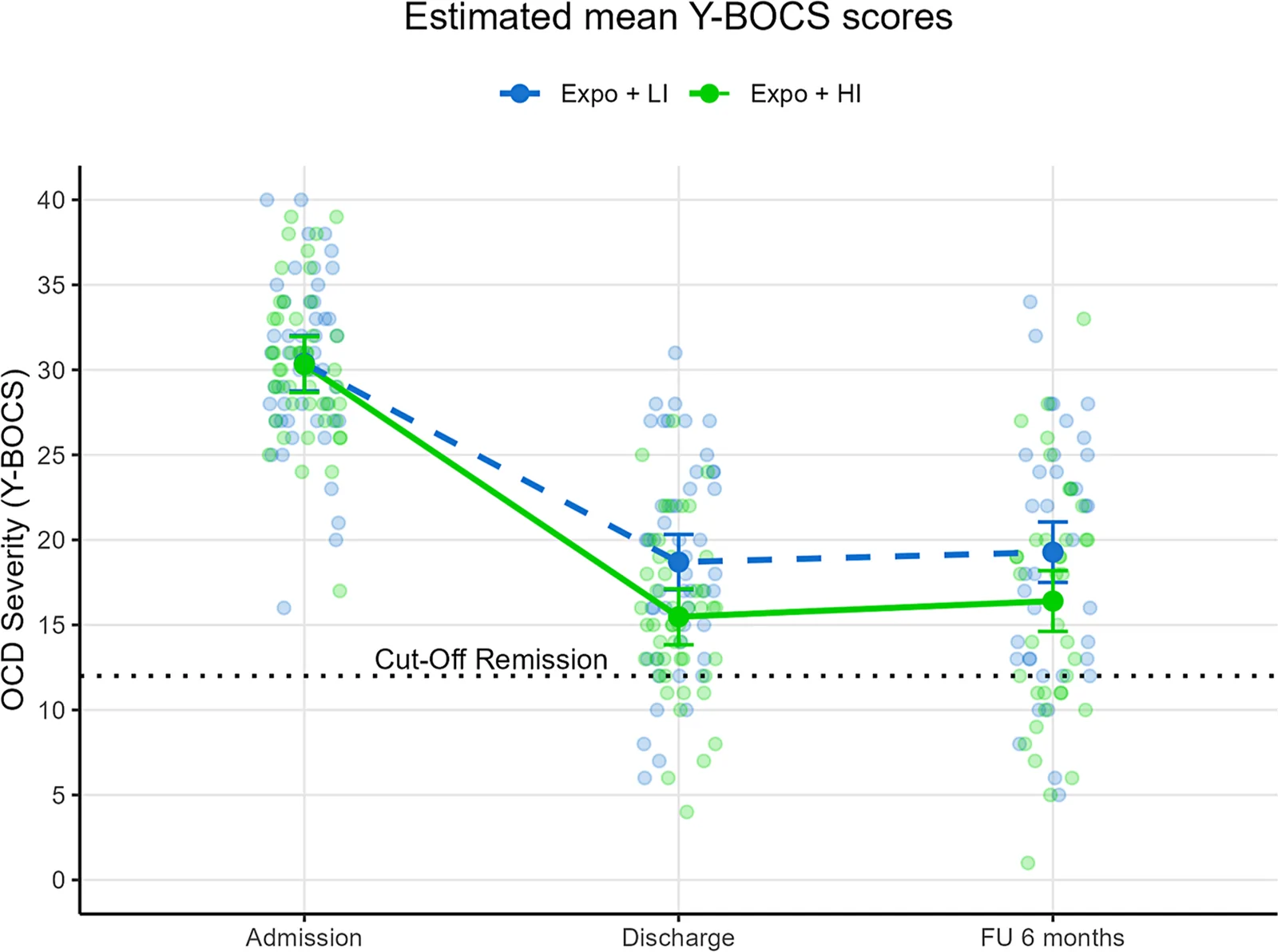
Estimated mean Y-BOCS scores in the high-intensity exercise (ExpoHI) and low-intensity control (ExpoLI) groups at admission, discharge, and 6-month follow-up. Error bars represent 95% confidence intervals of the estimated marginal means. Individual data points are displayed, and the dotted horizontal line indicates the remission threshold (Y-BOCS ≤ 12).

**Table 2.** Mean scores, standard deviations, test statistics, and between-group effect sizes of the differences for primary and secondary outcomes of the ITT sample (*N* = 97) between ExpoHI and ExpoLI

​	ExpoHI (*n* = 48)		ExpoLI (*n* = 49)		Group difference		
	M (SD)	*n*	M (SD)	*n*	Est. M (95% CI)	*p*	*Cohen´s d* (95% CI)
Primary outcome							
Y-BOCS	​	​	​	​	Model 0	​	​
Baseline	30.31 (4.32)	48	30.39 (5.02)	49	–	–	–
Post-treatment	15.48 (4.81)	48	18.69 (6.07)	49	−3.14 (−5.47, −0.81)	**0.009**	−0.76 (−1.33, −0.19)
FU 6 months	16.24 (7.08)	38	18.95 (7.33)	38	−2.80 (−5.34, −0.26)	**0.030**	−0.68 (−1.30, −0.06)
​	​	​	​	​	Model 1	​	​
Post-treatment	–	–	–	–	−3.14 (−5.47, −0.81)	**0.009**	−0.76 (−1.33, −0.19)
FU 6 months	–	–	–	–	−2.81 (−5.34, −0.27)	**0.030**	−0.68 (−1.30, −0.07)
Secondary outcome							
BDI							
Baseline	25.02 (11.30)	48	26.27 (10.86)	49	–	–	–
Post-treatment	17.07 (11.74)	44	19.26 (11.06)	47	−0.82 (−4.92, 3.29)	0.695	–
FU 6 months	15.21 (9.75)	38	22.53 (13.12)	38	−4.76 (−9.14, −0.38)	**0.033**	−0.67 (−1.29, −0.05)
PHQ-Anxiety							
Baseline	12.55 (5.10)	47	13.02 (4.60)	48	–	–	–
Post-treatment	8.27 (5.43)	44	9.54 (5.17)	46	−0.72 (−2.49, 1.05)	0.423	–
PHQ-Depression							
Baseline	13.30 (6.09)	47	13.92 (5.84)	48	–	–	–
Post-treatment	9.91 (5.69)	44	10.00 (6.39)	46	0.78 (−1.23, 2.79)	0.444	–
PHQ-Soma							
Baseline	11.30 (5.41)	47	10.81 (5.50)	48	–	–	–
Post-treatment	8.41 (4.99)	44	9.15 (5.77)	46	−1.11 (−2.77, 0.55)	0.189	–
BSI-GSI							
Baseline	72.26 (10.88)	47	73.24 (8.48)	49	–	–	–
Post-treatment	63.57 (14.17)	44	66.40 (11.24)	47	−1.63 (−5.16, 1.90)	0.362	–

Y-BOCS, Yale-Brown Obsessive-Compulsive Scale; BDI, Beck Depression Inventory; PHQ, Patient Health Questionnaire; BSI-GSI, Brief Symptom Inventory – Global Severity Index. Estimated mean group differences and p values are model-based difference-in-differences estimates from intent-to-treat analysis. Bold indicates p < 0.05. Cohen’s d based on model-based estimates. Model 0 includes time, treatment condition, and their interaction with a random intercept; Model 1 additionally adjusts for age, sex, comorbidity and medication.

### Secondary Clinical Outcomes

For the BDI–II, a significant time × condition interaction was observed at the 6-month follow-up, indicating a stronger reduction in depressive symptoms in the experimental group (Table 2). A significant between-group difference was found for the BSI phobic anxiety subscale (BSI-PHOB) at discharge, reflecting a stronger reduction in phobic anxiety symptoms in the experimental group. All other BSI and PHQ-D subscales showed no significant time × condition interactions.

### Exploratory Responder and Remission Rates

In exploratory analyses, responder rates (≥35% reduction in Y-BOCS scores) were higher in the experimental than in the control condition at discharge but not at the 6-month follow-up. Remission rates (Y-BOCS ≤12) did not differ significantly between the conditions at either time point. The detailed results are presented in Table 3. Rates were calculated using the denominators observed at each assessment point.

**Table 3.** Treatment response and remission by treatment conditions of the ITT sample (N = 97)

Variables	ExpoHI		ExpoLI		*p* value
	%	*n*	%	*n*	
Response					
Post-treatment	85.4	48	61.2	49	**0.01**
FU 6 months	71.1	38	50.0	38	0.09
Remission					
Post-treatment	22.9	48	16.3	49	0.45
FU 6 months	36.8	38	21.1	38	0.21

Response was defined as a ≥35% reduction from baseline Y-BOCS total score and remission as a Y-BOCS total score ≤12. Between-group differences were tested using Fisher’s exact test. Bold values indicate *p* < 0.05.

### Physical Exercise Parameters

Exercise parameters were analyzed within the experimental group using linear mixed-effects models, with time (admission vs. discharge) as a fixed effect and random intercepts for participants. The random slopes did not improve the model fit and were not retained. No significant change was observed in the heart rate (F = 0.54, *p* = 0.468; Δ = −3.42; dz = −0.12) or blood lactate level (F = 1.11, *p* = 0.296; Δ = 3.49; dz = 0.14) at the IANS. The power output at the IANS increased significantly from admission to discharge (F = 5.08, *p* = 0.031; Δ = 8.05; dz = 0.40), whereas the maximum relative power output did not change significantly (F = 1.98, *p* = 0.165; Δ = 0.61; dz = 0.22).

### Harms and Deterioration

No injuries, cardiovascular complications, or intervention-related dropout were observed in any group. One participant in the ExpoHI group reported transient muscle soreness following a training session; however, the participant completed regular inpatient sports activities on the same day. No participant showed worsening OCD symptom severity from baseline to discharge. At the 6-month follow-up, worsening relative to baseline was observed in only 4 of 76 participants (5.3%), equally distributed across treatment conditions (2 participants in each group).

## Discussion

This randomized controlled trial investigated whether post-exposure high-intensity aerobic exercise improves treatment outcomes compared with an active post-exposure control condition in patients with OCD. The results showed significantly greater reductions in obsessive-compulsive symptoms in the high-intensity exercise group than in the low-intensity active control group. These differences were evident at discharge and remained significant at the 6-month follow-up. In addition, the responder rates were significantly higher in the exercise condition at discharge (85.4% vs. 61.2%). Secondary analyses further indicated significantly greater reductions in depressive symptoms at the 6-month follow-up in the ExpoHI group. Taken together, these findings provide the first randomized controlled evidence that high-intensity aerobic exercise administered immediately after exposure produced greater reductions in obsessive-compulsive symptoms than an active post-exposure control condition in patients with OCD.

The magnitude of the treatment effects observed in the present study was comparable to those reported for other anxiety-related disorders. The between-group effect size for OCD symptom reduction was Cohen’s *d* = 0.76 at discharge and d = 0.68 at the 6-month follow-up. Similarly, Bryant et al. [3] reported an augmentation effect of aerobic exercise combined with exposure therapy in patients with post-traumatic stress disorder of approximately d ≈ 0.60 at the 6-month follow-up, while Gaudlitz et al. [42] reported a comparable effect (d ≈ 0.66) following aerobic exercise as an adjunct to cognitive behavioral therapy in panic disorder. Although controlled studies examining exercise augmentation in OCD remain scarce, a recent systematic review and meta-analysis reported large pre-post reductions in OCD symptom severity following exercise interventions (Hedges’ g = 1.33), accompanied by moderate improvements in anxiety and depression symptoms [22]. However, the authors also noted that the available evidence was based on a limited number of trials with a moderate-to-high risk of bias. Therefore, the present randomized trial extends the current evidence by demonstrating an additional treatment effect of high-intensity post-exposure exercise compared with the active control condition within an intensive multimodal OCD treatment program.

Although the present study does not permit conclusions regarding the mechanisms underlying the observed treatment differences, several plausible explanations warrant further consideration. One possible explanation relates to extinction learning, which underlies exposure therapy. Experimental evidence suggests that acute exercise may influence the consolidation of extinction memory. However, in a fear-conditioning study, vigorous exercise after extinction learning increased physiological arousal but did not selectively enhance extinction memory; instead, stronger fear responses occurred when extinction learning was incomplete [28]. These findings do not support a straightforward extinction-consolidation account and suggest that post-exposure exercise could potentially be counterproductive when extinction learning is incomplete. Another explanation is that exercise may have promoted meaningful, health-oriented behavioral engagement that counteracted compulsive routines and avoidance patterns, particularly given that individuals with OCD often show poorer somatic health and less healthy lifestyle habits [43]. In addition, the period immediately following the ERP may represent a clinically relevant phase during which obsessive distress and cognitive-emotional processing remain active. Acute exercise administered during this period could plausibly have influenced ongoing affective or cognitive processes, for example, through behavioral activation, transient mood enhancement, attentional distraction, or changes in physiological arousal. These alternative explanations cannot be distinguished in the present study and should be examined in future mechanistic studies. Accordingly, the present design cannot determine whether the observed benefits reflect a specific enhancement of exposure learning or more general therapeutic effects of acute exercise.

From a clinical perspective, aerobic exercise represents a simple, scalable, low-risk adjunct that can be readily integrated into exposure-based treatment programs. Given the substantial nonresponse rates in OCD, structured post-exposure exercise may provide a practical, non-pharmacological addition to established therapeutic protocols. Notably, the additional benefit observed in the present study was achieved within an intensive multimodal inpatient treatment program in which standard treatment already produces large overall effects (Hedges’ g = 1.34) [19]. Nevertheless, the clinical significance of the observed between-group differences remains uncertain. The adjusted difference in symptom change was −3.14 points (95% CI [−5.47, −0.81]) at discharge and −2.81 points (95% CI [−5.34, −0.27]) at the 6-month follow-up; however, no independently validated minimally important difference has been established specifically for the self-report Y-BOCS used in the present study. Moreover, remission rates did not differ significantly between treatment conditions. Accordingly, these findings should be considered preliminary and require replication in larger trials designed to disentangle exercise-specific and nonspecific treatment effects. Furthermore, it remains unclear whether the observed effects generalize to routine outpatient care, where treatment intensity, supervision, and adherence are likely to differ substantially from inpatient settings. Future studies should therefore examine whether repeated implementation of post-exposure high-intensity exercise over longer treatment periods, particularly in outpatient ERP, yields larger cumulative benefits and translates into clinically meaningful improvements in functioning, quality of life, and long-term outcomes.

Several limitations of this study should be considered when interpreting the findings. First, this study was conducted in a single specialized inpatient setting. Participants and therapists could not be blinded to treatment allocation because of the nature of the intervention, and OCD symptom severity was assessed using the self-report version of the Y-BOCS rather than the clinician-administered version, which remains the reference standard in OCD treatment trials. Furthermore, the responder and remission criteria were originally developed for the clinician-administered Y-BOCS and should therefore be interpreted cautiously when applied to the self-report version. Although participants were informed that two post-exposure interventions were being compared without disclosure of a superiority hypothesis, treatment expectancy and intervention credibility were not formally assessed. Consequently, differential expectancy effects and demand characteristics cannot be excluded and may have contributed to the observed between-group differences in this study. In addition, pharmacological treatment was not controlled, and medication was partially initiated or adjusted during inpatient treatment, which may have influenced the clinical outcomes. Importantly, the observed treatment effects remained stable after adjusting for psychopharmacological medication changes, suggesting that the superiority of ExpoHI is unlikely to be attributable to differential medication management between treatment conditions. Finally, the follow-up period was limited to 6 months, and longer-term outcomes remain to be investigated.

Future studies should replicate these findings in pragmatic multicenter trials and outpatient settings to establish their generalizability. In addition, studies are needed to clarify the mechanisms underlying the observed treatment effects by combining clinical outcomes with measures of extinction learning, behavioral activation, treatment expectancy, and biological markers where appropriate. Ecological momentary assessment may further help characterize cognitive-emotional processes during the hours following ERP and determine whether acute exercise influences distress trajectories, compulsive urges, perseverative thinking, affect regulation, or daily functioning. Future studies should examine whether the timing of exercise relative to ERP influences treatment outcomes. Whereas the present intervention was administered immediately after exposure to target the post-learning consolidation period, previous work in panic disorder applied aerobic exercise before exposure [42], suggesting that different temporal windows may engage distinct mechanisms and should be directly compared. In addition, the dose-response relationships between exercise intensity, duration, and treatment outcomes, as well as patient characteristics associated with the greatest benefit, should be systematically examined. Finally, pragmatic implementation studies are needed to evaluate the feasibility and effectiveness of digitally supported or home-based post-exposure exercise interventions in routine outpatient care.

## Conclusion

In summary, high-intensity aerobic exercise performed immediately after exposure therapy produced greater reductions in obsessive-compulsive symptoms than an active post-exposure comparison intervention in patients receiving intensive inpatient treatment. Although the mechanisms underlying this effect and its clinical significance require further investigation, these findings provide preliminary evidence that post-exposure aerobic exercise may represent a feasible adjunct to exposure-based treatment. Replication in larger pragmatic multicenter and outpatient trials is warranted before widespread clinical implementation can be recommended.

## Acknowledgments

The authors thank the clinical staff of the OCD specialty ward at the Schoen Clinic Roseneck for their support in patient recruitment and study implementation.

## Statement of Ethics

Ethical approval was granted by the Ethics Committee of Ludwig-Maximilians-Universität Munich (Reference No. 24-0325), and the study adhered to the principles of the Declaration of Helsinki. All participants provided written informed consent. The study was prospectively registered in the Open Science Framework (OSF; https://osf.io/6u7cv/; registration DOI: 10.17605/OSF.IO/PK2ZM; registered on August 21, 2024).

## Conflict of Interest Statement

Mark Mühlbacher has reported no financial interests. Non-financial interests include employment at Schoen Clinic Roseneck, Prien, Germany, and membership in the German Society for Obsessive-Compulsive Disorders (DGZ e.V.) and the German Association for Psychiatry, Psychotherapy, and Psychosomatics (DGPPN). Rebecca Schennach has no financial interests. Non-financial interests include employment as senior consultant psychiatrist at Schoen Clinic Roseneck, Germany, membership in the DGPPN and DGPM, and full membership in the International Society of Schema Therapy (ISST). Thomas Bösl has no financial interests. Non-financial interests include employment at Schoen Clinic in Roseneck, Germany. Stefan Koch has no financial interest. Non-financial interests include employment at the Schoen Clinic Roseneck, Germany. Christoph Bamberg has reported no financial interests. Non-financial interests include employment in the Department of Psychiatry and Psychotherapy, University Hospital Munich (Science Office). Ulrich Voderholzer reports financial interests related to the FIAT study (“Family-based telemedical versus institutional therapy for anorexia nervosa”), funded by the Innovation Fund of the Federal Republic of Germany. He also received honoraria for lectures and educational activities from scientific societies and continuing education providers (nonindustry). Non-financial interests include his role as a Medical Director and Head Physician at Schoen Clinic Roseneck in cooperation with the Department of Psychiatry and Psychotherapy, University Hospital Munich (Science Office); Chair of the Scientific Advisory Board of the DGZ e.V.; Vice Chair of the DGZ e.V.; Head of the German S3 Guideline Commission for Obsessive-Compulsive Disorders of the DGPPN; member of the DGPPN Psychotherapy Committee; Chair of the Eating Disorders Section of the World Psychiatric Association (WPA); spokesperson of the German Eating Disorders Association (BFE); Head of the Innovation, Science, and Research Working Group of the Psychosomatic Clinics of Schoen Clinic; and Head of the Science Office for Psychiatry, Psychosomatics, and Psychotherapy at Schoen Clinic Roseneck.

## Funding Sources

The authors declare no funding was obtained or used for this study.

## Author Contributions

M.M.: conception, study design, data acquisition, statistical analysis, data interpretation, critical revisions, first draft of the manuscript, and training and supervision of the research assessors. R.S.: conception, study design, data acquisition, interpretation, and critical revision. C.B.: statistical analysis, data interpretation, and critical revisions. T.B.: conception, data interpretation, and critical revisions. S.K.: conception, study design, data interpretation, and critical revision. U.V.: conception, data interpretation, and critical revision.

## Data Availability

Data supporting the findings of this study are not publicly available because of their sensitive nature. Data sharing may be possible upon reasonable request from the corresponding author.
